# N_2_O decomposition properties of Ru catalysts supported on various oxide materials and SnO_2_

**DOI:** 10.1038/s41598-020-78744-x

**Published:** 2020-12-10

**Authors:** Satoshi Hinokuma, Takeshi Iwasa, Yoshihiro Kon, Tetsuya Taketsugu, Kazuhiko Sato

**Affiliations:** 1 Interdisciplinary Research Center for Catalytic Chemistry, National Institute of Advanced Industrial Science and Technology (AIST), Central 5-2, 1-1-1 Higashi, Tsukuba, Ibaraki 305-8565 Japan; 2 Department of Chemistry, Faculty of Science, Hokkaido University, Sapporo, 060-0810 Japan; 3 Institute for Chemical Reaction Design and Discovery (WPI-ICReDD), Hokkaido University, Sapporo, 001-0021 Japan; 4 PRESTO, Japan Science and Technology Agency, Kawaguchi, 332-0012 Japan

**Keywords:** Chemistry, Engineering, Materials science

## Abstract

Nitrous oxide (N_2_O) is a stratospheric ozone depleting greenhouse gas that has global warming potential. As the catalytic decomposition of N_2_O is one of the most promising techniques for N_2_O emissions abatement, in this study, for this purpose the properties of Ru supported on various oxide materials were investigated under excess O_2_ conditions, and the identities of the N_2_O adsorption species on the catalysts were confirmed. To clarify the correlation between the catalytic properties and N_2_O decomposition activity, the supported Ru catalysts were characterised by means of powder X-ray diffraction, X-ray fluorescence measurements, energy-dispersive X-ray mapping and several gas sorption techniques. The results showed that the redox properties for Ru (RuO_2_) at low temperature are closely associated with N_2_O decomposition activity. The local structures, optimal Ru loading and N_2_O adsorption species of the novel Ru/SnO_2_ catalysts were studied and they showed high activity for N_2_O decomposition.

## Introduction

N_2_O is a stratospheric ozone depleting greenhouse gas that has a long lifetime of approximately 114 years and a global warming potential that is 310 times higher than that of CO_2_^1–11^. In addition, recently, anthropogenic N_2_O emissions, from fossil fuel-using industries and biomass combustion, as well as from chemical plants that produce adipic and nitric acid, have been annually increasing^8^. If every country and/or international organisation in the world does not put in place mitigation strategies, N_2_O emissions are forecast to approximately double by 2050^8–11^. To overcome these issues, catalytic decomposition of N_2_O to N_2_ and O_2_ is one of the most promising and economical techniques for emissions abatement, because the N_2_O emissions from combustion and chemical plants can be controlled using only end-of-pipe technologies employing exhaust heat. Over the past few years, supported metal oxides, noble metals and/or metal oxides and composite oxide (perovskites, hydrotalcites, spinels and hexa-aluminates) catalysts for N_2_O decomposition have been studied^8,12–34^. In terms of supported catalysts, it has been shown that their activities for N_2_O decomposition follow the order of Ru, Rh, Ir > Pd > Cu > Fe > Pt > Ni > Mn^8^.

In the case of active Ru catalysts, as Ru species are efficient catalysts for the dissociation of the N–O bond in N_2_O, their catalysts are thus promising candidates for N_2_O decomposition^26–34^. However, the catalytic activities of Ru species are strongly related to their sizes, distributions and degrees of agglomeration. Zheng et al. studied the effect of support materials (MgO, SiO_2_, CeO_2_, Al_2_O_3_, TiO_2_, active carbon and SiC) on the catalytic properties of supported Ru for the N_2_O decomposition reaction^31^. They concluded that Ru/TiO_2_ shows high activity for N_2_O decomposition, where the activity was found to be related to the reducibility of the catalyst. Lin et al. also suggested that Ru supported on rutile-type TiO_2_ exhibited higher N_2_O decomposition activity compared with Ru/anatase-TiO_2_, Ru/Al_2_O_3_, Ru/SiO_2_ and other such systems, because its catalytic properties are induced by Ru metal dispersion and its monolayer structure^30^. Komvokis et al. prepared highly dispersed Ru/γ-Al_2_O_3_ via a conventional impregnation and ethylene glycol (EG) method and reported that Ru/γ-Al_2_O_3_ prepared using the EG method featured metallic Ru nanoparticles with a size of ca. 1–3 nm and high activity for N_2_O decomposition with H_2_O, SO_2_ and NO^28^. Recently, active Ru supported on perovskite-like La_1.6_Sr_0.4_NiO_4_ was prepared by Sui et al.^33^, who indicated that the activity arose due to the desorption of a large amount of oxygen from the active sites at low temperature and the ability of the oxygen vacancies to regenerate. Therefore, as stated above, Ru species are expected to be possible candidates for N_2_O decomposition catalysts due to their specific catalytic properties.

For N_2_O emissions from industrial combustion processes (i.e. fluidised bed combustion), it is considered that these emissions contain, amongst other gases, ca. 50–200 ppm of N_2_O, excess O_2_ (2–10%) and 10% H_2_O (water vapour)^8,35,36^. Therefore, as any catalysts that are developed may potentially be used in the decomposition of N_2_O from industrial combustion processes as a practical application, the evaluation of their catalytic properties under steady gas emissions conditions is required. Therefore, in this study, the N_2_O decomposition reaction properties of Ru supported on various oxide materials under excess O_2_ conditions were focused on, and the identities of the N_2_O adsorption species on these catalysts were confirmed. As the novel Ru/SnO_2_ catalysts in this work showed high activity for N_2_O decomposition, their local structures, optimal Ru loading and N_2_O adsorption species were determined. Finally, the reproducibility of the effects that H_2_O (water vapour) have on the N_2_O decomposition properties was evaluated.

## Results and discussion

### Ru supported on various metal oxide materials

**Figure 1:**
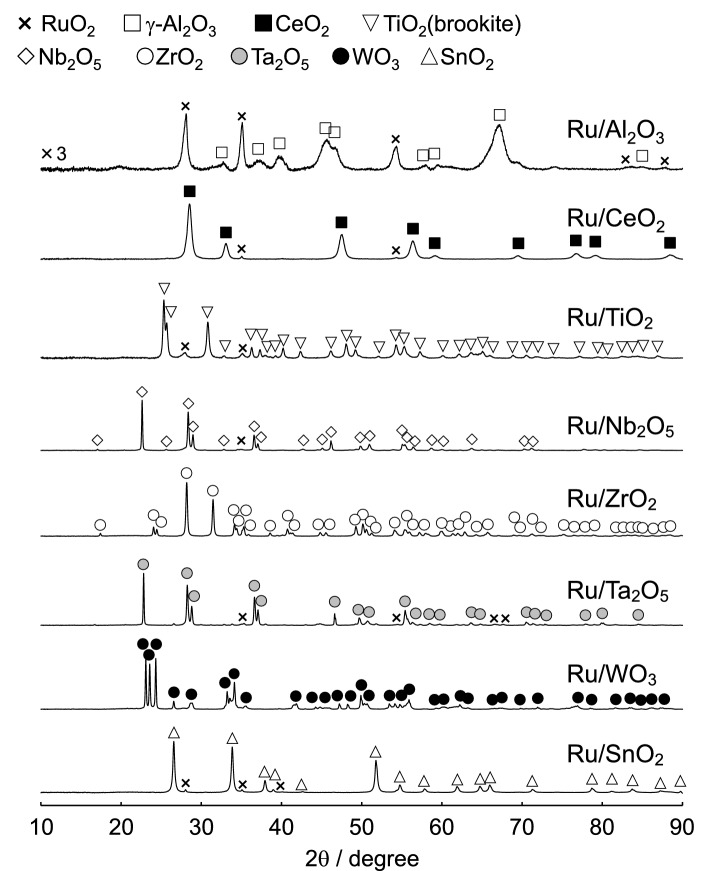
PXRD patterns of 5.0 wt% Ru supported on various different metal oxide materials prepared by impregnation, followed by drying and calcination at 600 °C for 3 h in air.

Figure 1 shows the powder X-ray diffraction (PXRD) patterns of Ru supported on various metal oxide materials. The diffraction peaks for the Ru of all of the supported catalysts could be assigned to RuO_2_, and the Al_2_O_3_ of Ru/Al_2_O_3_ and TiO_2_ of Ru/TiO_2_ could be assigned to γ phase and brookite structures, respectively. As the other support materials also had compositional formulas, the solid-state reaction of RuO_2_ and the support materials was not observed.

**Figure 2:**
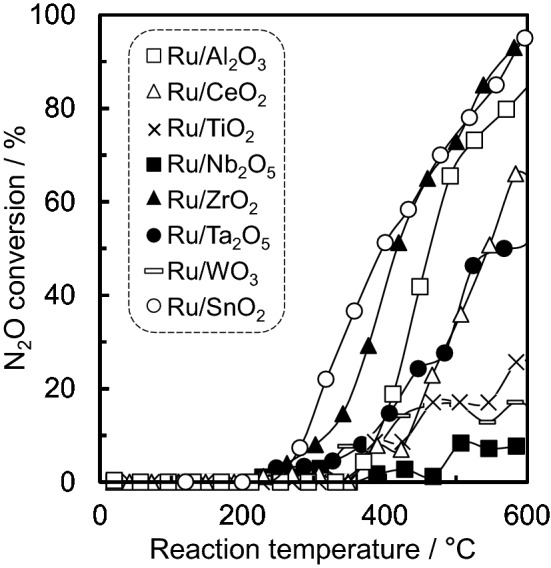
Catalytic activity for N_2_O decomposition reaction over 5.0 wt% Ru supported on various different metal oxide materials. Reaction conditions: 200 ppm of N_2_O, 10% O_2_ and N_2_ balance at 100 cm^3^ min^−1^ (W/F = 5.0 × 10^−4^ g·min cm^−3^).

Figure 2 shows a comparison of the temperature dependence of N_2_O conversion for the various different supported Ru catalysts. As none of the catalysts showed NO production, as detected by non-dispersive infrared (NDIR) spectroscopy, it was presumed that N_2_O decomposed into N_2_ and O_2_. For Ru/SnO_2_ and Ru/ZrO_2_, the light-off curves of N_2_O were obtained at approximately 200 °C, although the light-off temperature at which 90% conversion of N_2_O occurred was not reached for all catalysts at a reaction temperature of 600 °C. In the case of Ru/Nb_2_O_5_, it showed almost no activity.

Table 1 summarises the properties of the different catalysts, in which the activity is expressed in terms of the light-off temperature at which 50% conversion of N_2_O was achieved (*T*_50_). The *T*_50_ values increased in the order of SnO_2_ < ZrO_2_ < Al_2_O_3_ < CeO_2_ < Ta_2_O_5_ < TiO_2_ ≈ WO_3_ ≈ Nb_2_O_5_, which bears no relation to the Brunauer − Emmett − Teller surface area (S_BET_) values of their catalysts. The order of the *T*_50_ values is almost consistent with the order of the reduction temperatures observed from the H_2_-temperature-programmed reduction (TPR) experiments (see Table 1 and Supplementary Figure S1): Al_2_O_3_ < SnO_2_ < ZrO_2_ < CeO_2_ < Ta_2_O_5_ < TiO_2_ < WO_3_ < Nb_2_O_5_, suggesting that the redox properties for Ru (RuO_2_) at low reaction temperature are closely related to the N_2_O decomposition activity. Because, in addition, the supported Ru catalysts with lower reduction temperatures tended to exhibit higher metal dispersion (smaller particle size), it is considered that highly dispersed Ru (RuO_2_) particles act as an active catalyst for N_2_O decomposition reaction. Ru/CeO_2_ exhibited high metal dispersion (18%), but its activity showed medium. In case of CeO_2_-supported catalysts, the overestimation for the metal dispersion was previously reported, because of CO adsorbed on CeO_2_ support as carbonate species^37^. Therefore, it is suggested that the overestimation for the dispersion for Ru/CeO_2_ was also caused by CO adsorption on CeO_2_, and its relationship between the dispersion and activity for Ru/CeO_2_ are low. Ru3d XPS spectra for 5.0 wt% Ru catalysts supported on various oxide materials were obtained (Supplementary Figure S2). According to the previous report for Ru3d XPS spectra analysis^38^, the binding energies of Ru3d_5/2_ peaks for all catalysts could be assigned to the oxidation state of Ru^4+^ (approximately 280.4 eV), which is consistent with the assignment for RuO_2_ of XRD patterns. In addition, Ru3d_5/2_ peak area and intensity for Ru/SnO_2_ showed slightly higher in comparison with the other catalysts, which indicates that Ru surface concentration for Ru/SnO_2_ is higher in agreement with high Ru metal dispersion estimated by CO adsorption.

**Table 1:** Catalytic properties of 5.0 wt% Ru supported on various different oxide materials.

Catalyst	Phase	*T*_50_^[a]^/°C	*S*_BET_/m^2^ g^−1^	*T*_red._^[b]^/°C	Metal dispersion^[c]^*/*%	Desorbed gas ^[d]^ */*mmol g^−1^	
						NH_3_	NO
Ru/Al_2_O_3_	RuO_2_/γ-Al_2_O_3_	454	137	70	12	0.264	0.040
Ru/CeO_2_	RuO_2_/CeO_2_	531	64	156	18	0.017	0.031
Ru/TiO_2_	RuO_2_/TiO_2_(brookite)	–	41	191	2	0.173	0.040
Ru/Nb_2_O_5_	RuO_2_/Nb_2_O_5_	–	10	237	1	0.028	0.016
Ru/ZrO_2_	RuO_2_/ZrO_2_	420	11	128	9	0.044	0.037
Ru/Ta_2_O_5_	RuO_2_/Ta_2_O_5_	577	4	160	3	0.014	0.024
Ru/WO_3_	RuO_2_/WO_3_	–	10	194	2	0.066	0.019
Ru/SnO_2_	RuO_2_/SnO_2_	395	14	102	7	0.033	0.029

[a] Temperature at which N_2_O conversion reached 50%. [b] Temperature of the first H_2_ consumption peak determined by H_2_-TPR. [c] Calculated with the stoichiometric adsorption of CO on Ru in a ratio of CO:Ru = 1:1. [d] Estimated using NH_3_- and NO-TPD in the temperature range of 100–500 °C.

To study the acid and base properties of the supported Ru catalysts, the amount of desorbed gas per weight on the catalyst was estimated using NH_3_- and NO-temperature-programmed desorption (TPD) and was found to be in the range 100–500 °C (see Supplementary Figure S3 and Figure S4 for more details), the data of which are also summarised in Table 1. Ru/Al_2_O_3_ and Ru/TiO_2_ showed high amounts of desorbed NH_3_, whereas Ru/CeO_2_ exhibited a low amount despite it having a relatively high S_BET_ value. The other catalysts also showed lower acidity than Ru/Al_2_O_3_. However, in terms of NO desorbability, there was no significant difference as well as no relation to the S_BET_ value. However, it was also implied that the supported Ru with a higher amount of NO desorbability tended to approximately exhibit higher N_2_O decomposition activity, which therefore indicated that there is almost a correlation between the base properties and the catalytic N_2_O decomposition activity.

**Figure 3:**
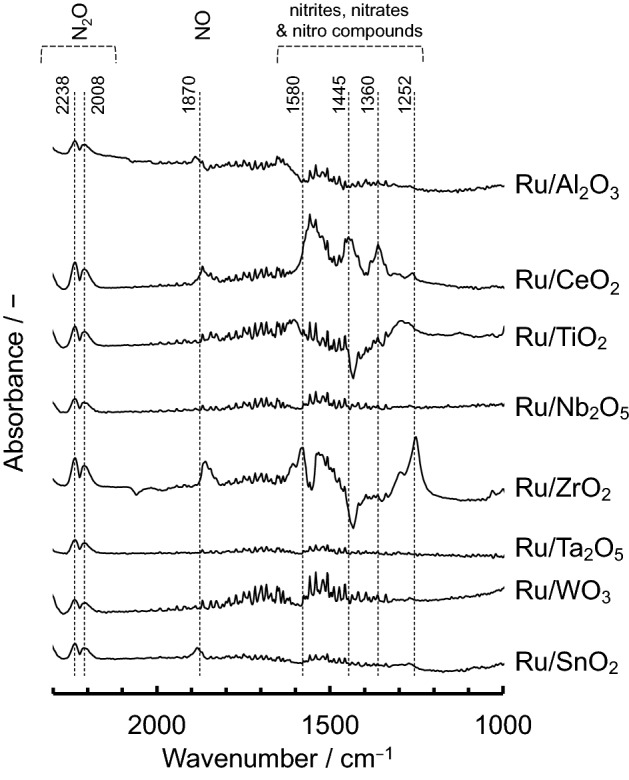
In situ FTIR spectra of N_2_O adsorbed on 5.0 wt% Ru supported on various different metal oxide materials, measured at 200 °C in gas feeds of 200 ppm of N_2_O and N_2_ balance.

To confirm the identities of the N_2_O adsorption species on the various different supported Ru catalysts and the reasons for their different activities, in situ Fourier-transform infrared (FTIR) spectra were recorded at 200 °C, which is the approximate initiation temperature for N_2_O decomposition (Fig. 3). Based on previous reports^32,39–42^, two bands at 2238 and 2008 cm^−1^, attributed to adsorbed N_2_O, were observed for all of the catalysts. Several other bands were also observed in the range of 1000–1700 cm^−1^, which were attributed to nitrites, nitrates and nitro compounds that were adsorbed on the catalysts. These bands determinably appeared for Ru/CeO_2_ and Ru/ZrO_2_, which is consistent with the high NO adsorbability (basicity) in the range of 300–600 °C (see Supplementary Figure S4 for more details). In addition, the band at approximately 1870 cm^−1^ attributed to adsorbed NO was observed for Ru/Al_2_O_3_, Ru/CeO_2_, Ru/ZrO_2_ and Ru/SnO_2_, which showed that these systems have relatively high activity for N_2_O decomposition. Therefore, it was concluded that this band attributed to NO species can be considered as belonging to activated N_2_O.

### Effects of Ru loading on catalytic N_2_O decomposition

**Figure 4:**
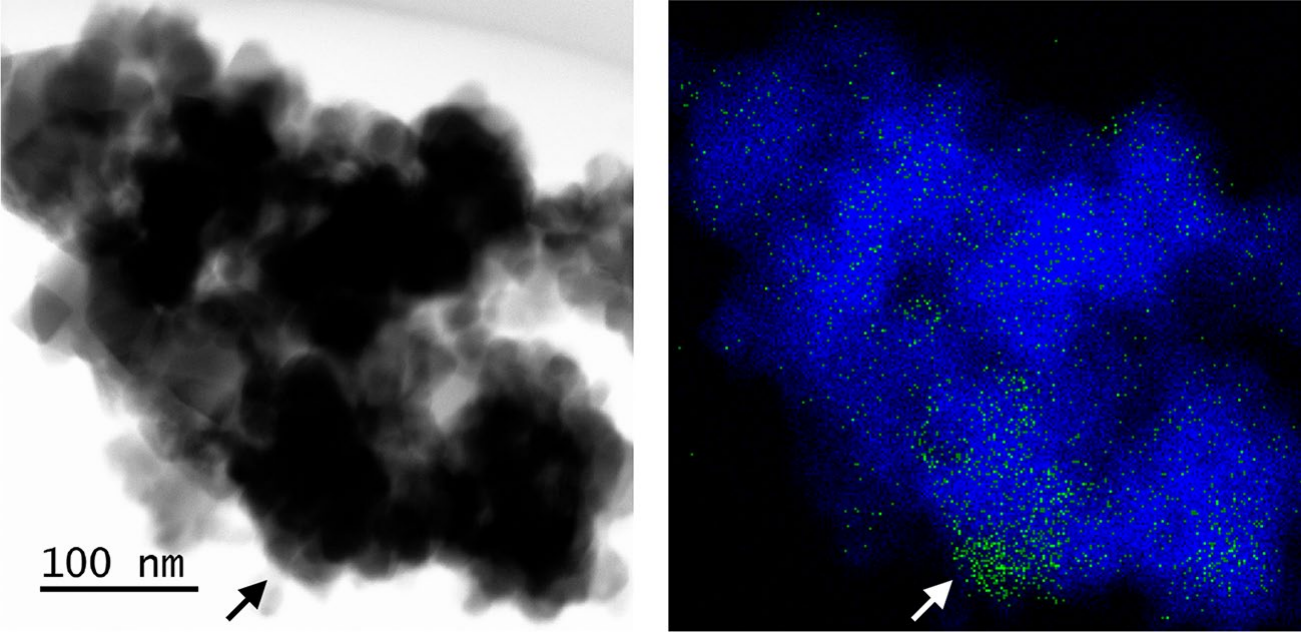
(left) TEM image and (right) EDX mapping analysis of 5.0 wt% Ru/SnO_2_. The blue and green dots correspond to the Sn − L and Ru − K fluorescence lines, respectively.

As the Ru/SnO_2_ catalyst exhibited high N_2_O decomposition activity, the optimal amount of Ru loading, its local structure and catalytic properties were comprehensively studied. In the PXRD patterns of the catalysts with different loading amounts of Ru (see Supplementary Figure S5 for more details), the diffraction peaks for SnO_2_ can be observed for all of the catalysts, whereas the diffraction peaks for RuO_2_ can be observed in the patterns for the catalysts with a Ru loading of higher than 5.0 wt%. In addition, the intensities of the diffraction peaks of RuO_2_ increased upon an increase in the Ru loading, which probably suggests an increase in the crystallinity and/or particle size upon the increase in RuO_2_. In terms of the S_BET_ values of the catalysts with different amounts of Ru loading (see Supplementary Table S1 for more detail), the values were observed to decrease upon an increase in the Ru loading, probably because of the lower proportion of SnO_2_ to a higher proportion of RuO_2_. The local structure of the 5.0 wt% Ru/SnO_2_ sample was characterised using scanning transmission electron microscopy and energy-dispersive X-ray mapping (STEM-EDX) mapping (Fig. 4). The bright-field STEM image revealed SnO_2_ particles present in the sample with sizes of approximately 10–100 nm. From the overlay image of the EDX mapping, Sn − L (blue) and Ru − K (green) fluorescence lines can be observed. Therefore, based on these and the PXRD results, RuO_2_ particles with a size of approximately 50 nm, as shown by the arrows in Fig. 4, were dispersed on the SnO_2_ support.

**Figure 5:**
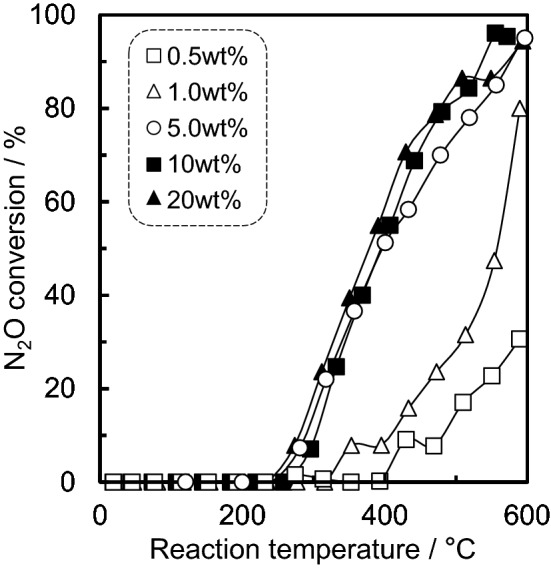
Catalytic activity for the N_2_O decomposition reaction over 0.5–20 wt% Ru/SnO_2_. Reaction conditions: 200 ppm of N_2_O, 10% O_2_ and N_2_ balance at 100 cm^3^·min^−1^ (W/F = 5.0 × 10^−4^ g·min·cm^−3^).

In Fig. 5 and Supplementary Table S1 the temperature dependence of N_2_O conversion are compared for 0.5–20 wt% Ru/SnO_2_. It was found that 0.5 and 1.0 wt% Ru/SnO_2_ showed a lower activity of catalytic N_2_O decomposition, whereas 5.0–20 wt% Ru/SnO_2_ exhibited almost the same light-off profile for N_2_O and *T*_50_. Therefore, it was assumed that the optimal Ru loading supported on SnO_2_ was approximately 5.0 wt%. Supplementary Figure S6 also shows N_2_O conversion, NO selectivity and mass signal for N_2_O decomposition reaction over 5.0 wt% Ru/SnO_2_. NO selectivity was not observed. For the production of NO_2_, the mass signal for *m/z* value of 44 for N_2_O decreased, which is consistent with N_2_O conversion, whereas the mass signal for *m/z* value of 46 for NO_2_ was constant. In addition, we estimated the energy difference to generate NO_2_ from NO and O by density functional theory calculations at the level of BP86^43,44^/def-SV(P)^45^ under the resolution of identity approximation^46^ using TURBOMOLE^47^. Although NO_2_ can be formed barrierlessly from NO and O, the preparation of O from O_2_ requires large energy of 6.24 eV, which is larger than the NO_2_ formation energy from NO and O, 4.23 eV. Therefore, it is considered that not only NO but also NO_2_ were not produced during the N_2_O decomposition reaction over 5.0 wt% Ru/SnO_2_.

**Figure 6:**
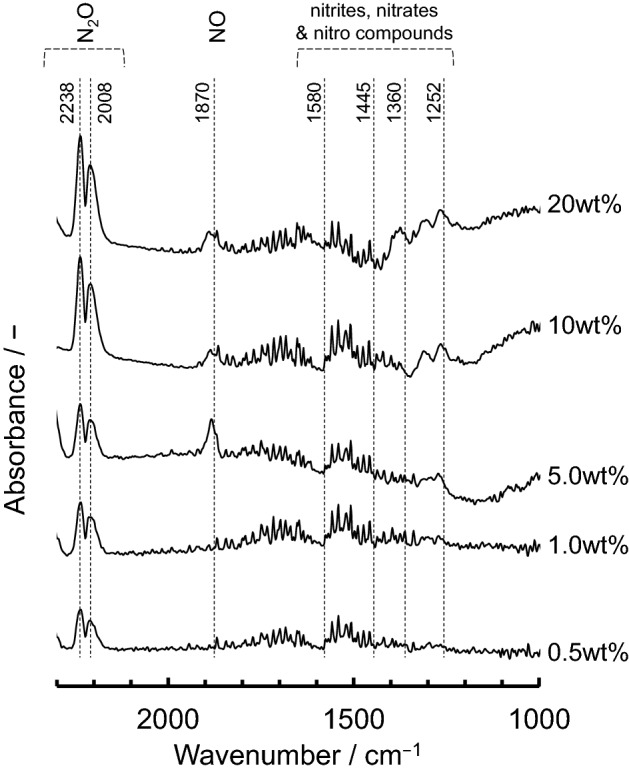
In situ FTIR spectra of N_2_O adsorbed on 0.5–20 wt% Ru/SnO_2_. The spectra were measured at 200 °C in gas feeds of 200 ppm of N_2_O and N_2_ balance.

To also confirm the relationship between the N_2_O adsorption species and decomposition activities, in situ FTIR spectra were recorded for 0.5–20 wt% Ru/SnO_2_ at 200 °C (Fig. 6). For all of the Ru/SnO_2_ samples, two bands were observed for adsorbed N_2_O, at 2238 and 2008 cm^−1^. However, bands for adsorbed NO (1870 cm^−1^) and NO_x_ compounds (1000–1700 cm^−1^) were present for the 5.0–20 wt% Ru/SnO_2_ that have high activities, which is consistent with the results that is Ru supported on the different metal oxide materials. Therefore, it is expected that this NO band can be considered as belonging to activated N_2_O. In situ FTIR spectra of N_2_O adsorbed on 5.0 wt% Ru/SnO_2_ at 300 °C and 400 °C were also confirmed (Supplementary Figure S7). The two bands attributed to adsorbed N_2_O (2008 and 2238 cm^−1^) were observed at 300 °C, whereas the bands were not observed at 400 °C. These bihaviors probably imply that catalytic N_2_O decomposition reaction was proceeded.

### Stability, reproducibility and the effects of water vapour

**Figure 7:**
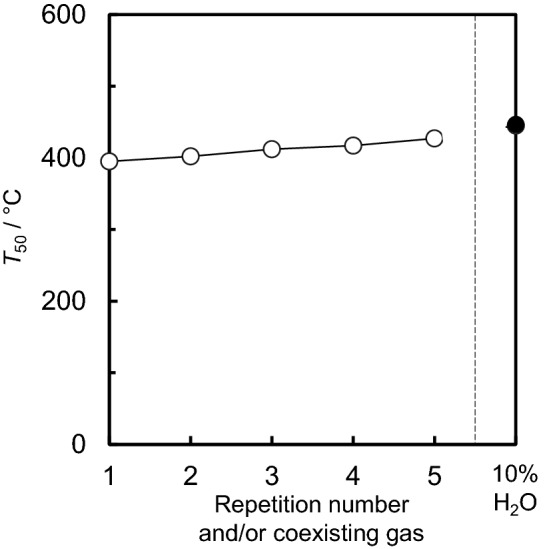
Catalytic activity (*T*_50_) for the N_2_O decomposition reaction over 5.0 wt% Ru/SnO_2_ as a function of the repetition number, and the effects of water vapour (10% H_2_O). Reaction conditions: 200 ppm of N_2_O, 10% O_2_ and N_2_ balance at 100 cm^3^·min^−1^ (W/F = 5.0 × 10^−4^ g min cm^−3^).

To evaluate the stability and reproducibility of the 5.0 wt% Ru/SnO_2_ catalyst, the N_2_O decomposition reaction was repeated five times. Figure 7, Supplementary Figure S8 and Supplementary Table S2 give summaries of the temperature dependence of N_2_O conversion for repeated cycles using 5.0 wt% Ru/SnO_2_. Upon repetition of the catalytic cycle, the light-off temperature and *T*_50_ tended to slightly increase. Because there is concern for the vaporisation as RuO_3_ and/or RuO_4_^48^, it is considered that the stability of RuO_2_ is low at high temperature. XRD patterns and Ru3d XPS spectra for 5.0 wt% Ru/SnO_2_ after N_2_O decomposition reaction were obtained (Supplementary Figure S9). For XRD pattens, the diffraction peaks for Ru/SnO_2_ after the reaction could be assigned to RuO_2_ and SnO_2_. In comparison with as-prepared catalysts (before reaction), not only the diffraction peaks but also Ru3d XPS spectra for 5.0 wt% Ru/SnO_2_ after reaction are almost no change. The Ru metal dispersion for 5.0 wt% Ru/SnO_2_ after N_2_O decomposition reaction at 600 °C was also estimated, and it showed 6%. In comparison with the dispersion for as-prepared 5.0 wt% Ru/SnO_2_ (7%), it is slight low. Therefore, it is suggested that the sintering of Ru (RuO_2_) was slightly induced by the N_2_O decomposition reaction at 600 °C. However, the light-off temperature at which 90% conversion was achieved was almost reached for all of the catalysts at 600 °C. Therefore, the stability and reproducibility of 5.0 wt% Ru/SnO_2_ were confirmed. In addition, time-on-stream stability of catalytic activity for the N_2_O decomposition reaction over 5.0 wt% Ru/SnO_2_ at 400 °C was also studied (Supplementary Figure S10). Because the stable N_2_O conversition (ca. 55%) was confirmed in approximately 2 h, it is considered that the the catalytic stability for 5.0 wt% Ru/SnO_2_ was verified.

The effects of water vapour (10% H_2_O) on the N_2_O decomposition reaction over as-prepared 5.0 wt% Ru/SnO_2_ were also evaluated (see the plots in Supplementary Figure S8). The N_2_O conversion profile for the reaction with H_2_O shifted to a higher temperature than that without H_2_O. In comparison with the *T*_50_ values for the N_2_O decomposition reaction carried out in the absence of H_2_O, the *T*_50_ values of that carried out in the presence of H_2_O was higher, at 50 °C (see Supplementary Table S2 for more details). Therefore, it could be deduced that the deactivation of 5.0 wt% Ru/SnO_2_ and/or inhibition of N_2_O adsorption on 5.0wt % Ru/SnO_2_ was induced by the presence of H_2_O.

## Conclusion

Ru catalysts supported on various different metal oxides were prepared by impregnation to evaluate their decomposition properties for N_2_O, which is a powerful greenhouse gas that is present in industrial emissions. From the PXRD patterns, the diffraction peaks for Ru of all of the supported catalysts could be assigned to RuO_2_. The *T*_50_ values were found to increase in the order of: SnO_2_ < ZrO_2_ < Al_2_O_3_ < CeO_2_ < Ta_2_O_5_ < TiO_2_ ≈ WO_3_ ≈ Nb_2_O_5_, which is almost consistent with the order of the reduction temperatures observed from the H_2_-TPR measurements. Therefore, it is considered that the redox properties for Ru (RuO_2_) at low reaction temperature are closely associated with N_2_O decomposition activity. In addition, according to the NO-TPD profiles and in situ FTIR data, a correlation can also be observed between the NO adsorption properties as well as the NO species considered to be activated N_2_O and the catalytic N_2_O decomposition activity. Among the supported Ru catalysts, Ru/SnO_2_ showed a high catalytic performance for the N_2_O decomposition reaction. SnO_2_ support materials induced the physicochemical properties, high reducibility (redox property), Ru (RuO_2_) dispersion and basicity for the catalysts, which are required for high N_2_O decomposition activity. As 5.0–20 wt% Ru/SnO_2_ exhibited almost the same light-off profiles for N_2_O and *T*_50_, the optimal amount of Ru loaded on the SnO_2_ support was found to be approximately 5.0 wt%. Although the deactivation of Ru/SnO_2_ was induced by H_2_O, the stability and reproducibility for N_2_O decomposition activity of Ru/SnO_2_ were confirmed.

## Methods

### Catalyst preparation

A wide variety of commercially available metal oxides, Al_2_O_3_ (JRC-ALO-8, Catalysis Society of Japan), CeO_2_ (JRC-CEO-5, Catalysis Society of Japan), TiO_2_, Nb_2_O_5_, ZrO_2_, Ta_2_O_5_, WO_3_ and SnO_2_ (Kojundo Chemical Lab.), were used as support materials for Ru. Supported Ru (5.0 wt% loading as Ru) samples were prepared via the impregnation of an aqueous solution of RuCl_3_ (Fujifilm Wako Pure Chemical Corporation), followed by drying and calcination at 600 °C for 3 h in air. To study the effects of Ru loading, 0.5–20 wt% of Ru/SnO_2_ catalysts that show a high performance in N_2_O decomposition were prepared in a similar manner for comparison.

### Characterisation

PXRD measurements were performed using mono-chromated Cu Kα radiation (40 kV, 15 mA, MiniFlex600, Rigaku). The chemical compositions of the samples were determined from X-ray fluorescence measurements (XRF, MESA-500 W, Horiba). Spectra from X-ray photoelectron spectroscopy (XPS) were obtained using Al Kα radiation (PHI 5000-VersaProbe, Ulvac-Phi). The C1s signal at 284.8 eV that was derived from adventitious carbon was used as a reference to correct for the effect of surface charge. STEM-EDX mapping were performed using a JEM-ARM200CF microscope (Jeol). S_BET_ calculations were performed using N_2_ adsorption isotherms, which were obtained at − 196 °C (ASAP2020, Micromeritics). H_2_-TPR measurements were performed in a flow system (5% H_2_/Ar) at a constant rate of 10 °C min^−1^ (Bel-cat, Microtrac-Bel). The NH_3_ and/or NO adsorbability of the catalysts were also studied through TPD. Prior to the measurements, the catalysts were treated at 500 °C for 1 h under an Ar flow and were subsequently cooled at 100 °C for 30 min in 5% NH_3_/Ar and 1% NO/Ar (50 cm^3^ min^−1^). After pre-treatment, the catalysts were heated to 500 °C under a He flow at a constant rate of 10 °C·min^−1^. The concentrations of the desorbed NH_3_ and/or NO in the effluent gas were analysed using an online thermal conductivity detector (TCD) signal (Bel-cat, Microtrac-Bel). The Ru metal dispersion was determined by pulsed CO chemisorption at 50 °C (Bel-metal, Microtrac-Bel) after the catalyst was oxidised using O_2_ and subsequently reduced using H_2_ at 400 °C. The metal dispersion was calculated from the molar ratio of chemisorbed CO to loaded Ru by assuming that the chemisorption stoichiometry of Ru:CO was 1:1.

### Catalytic N_2_O decomposition tests

The catalytic decomposition of N_2_O was performed in a flow reactor at atmospheric pressure. Catalysts (10–20 mesh, < 0.3 mm thickness, 50 mg) were fixed in a quartz tube (outside diameter: 6 mm, inside diameter: 4 mm) with quartz wool at both ends of the catalyst bed. The temperature dependence of the catalytic activity was evaluated by heating the catalyst bed from room temperature to 600 °C at a constant rate of 10 °C min^−1^ while a gas mixture containing 200 ppm of N_2_O, 10% O_2_ and N_2_ balance was supplied at 100 cm^3^ min^−1^ (W/F = 5.0 × 10^−4^ g min cm^−3^). For the catalysts that exhibited high performance, the reactions were repeated to evaluate the stability of the catalysts and the reproducibility of the results. Effects of water vapour (10% H_2_O) on the N_2_O decomposition reaction were also evaluated. The N_2_O, NO and gas concentrations were analysed using an NDIR analyser (VA-3011, Horiba) and quadrupole mass spectrometer (PrismaPlus, Pfeiffer).

In situ FTIR spectra were recorded on a FT/IR-6600 spectrometer (Jasco) using a diffuse-reflectance reaction cell with a BaF_2_ window connected to a gas supply and a heating system to enable measurements to be conducted under atmospheric pressure. First, the catalysts were preheated in situ in a flow of Ar at 400 °C for 30 min prior to each experiment. After pre-treatment, the temperature of the catalyst was decreased to 200 °C, followed by the subsequent purging of the cell with Ar and then filling with 200 ppm of N_2_O/N_2_ gas. Finally, FTIR spectra were recorded while the catalysts were maintained under a stream of N_2_O/N_2_.

## Supplementary information

Supplementary Information.
