# Supplementary material for: N2O decomposition properties of Ru catalysts supported on various oxide materials and SnO2
Source: Sci Rep. 2020 Dec 10;10:21605. doi: 10.1038/s41598-020-78744-x (PMC7728819; doi:10.1038/s41598-020-78744-x)
Supplement: Supplementary file 1 — Supplementary Information. [file 41598_2020_78744_MOESM1_ESM.docx]

Supplementary Information for

N_2_O Decomposition Properties of Ru Catalysts Supported on Various Oxide Materials and SnO_2_

Satoshi Hinokuma,^1,^* Takeshi Iwasa,^2,3,4^ Yoshihiro Kon,^1^ Tetsuya Taketsugu,^2,3^ and Kazuhiko Sato^1^

*^1^ Interdisciplinary Research Center for Catalytic Chemistry, National Institute of Advanced Industrial Science and Technology (AIST), Central 5-2, 1-1-1 Higashi, Tsukuba, Ibaraki 305-8565, Japan*

*^2^ Department of Chemistry, Faculty of Science, Hokkaido University, Sapporo 060-0810, Japan*

*^3^ Institute for Chemical Reaction Design and Discovery (WPI-ICReDD), Hokkaido University, Sapporo 001-0021, Japan*

*^4^ PRESTO, Japan Science and Technology Agency, Kawaguchi 332-0012, Japan*

Corresponding author:
Dr. Satoshi Hinokuma
E-mail: hinokuma-s@aist.go.jp
Tel: +81-29-849-1698

**Materials and Methods**

**Characterisation**

Powder X-ray diffraction (PXRD) measurements were performed using mono-chromated Cu Kα radiation (40 kV, 15 mA, MiniFlex600, Rigaku). Temperature-programmed reduction by H_2_ (H_2_-TPR) measurements were performed in a flow system (5% H_2_/Ar) at a constant rate of 10°C·min^−1^ (Bel-cat, Microtrac-Bel). The NH_3_ and/or NO adsorbability of the catalysts were also studied through temperature-programmed desorption (TPD). Prior to the measurements being made, the catalysts were treated at 500°C for 1 h under an Ar flow and subsequent cooling at 100°C for 30 min in 5% NH_3_/Ar and 1% NO/Ar (50 cm^3^·min^−1^). After pre-treatment, the catalysts were heated to 500°C under a He flow at a constant rate of 10°C·min^−1^. The concentrations of the desorbed NH_3_ and/or NO in the effluent gas were analysed using an online thermal conductivity detector (TCD) signal (Bel-cat, Microtrac-Bel).

**Catalytic N_2_O Decomposition Tests**

For catalytic N_2_O decomposition, the N_2_O conversion was calculated using the following formula:

N_2_O conversion: ([N_2_O_in_] − [N_2_O_out_]) / (N_2_O_in_) × 100

where (N_2_O_in_) is the inlet N_2_O concentration (200ppm) and (N_2_O_out_) is the outlet N_2_O concentration.

**Table S1.** Catalytic properties of the different loading amounts for the Ru/SnO_2_ catalysts.

| Catalyst | Phase | *T*_50_ | *S*_BET_ |
| --- | --- | --- | --- |
|  |  |  |  |
|  |  | ^[a]^ */*°C | /m^2^ g^−1^ |
| 0.5 wt% Ru/SnO_2_ | - | - | 16 |
| 1.0 wt% Ru/SnO_2_ | - | 571 | 16 |
| 5.0 wt% Ru/SnO_2_ | RuO_2_/SnO_2_ | 395 | 14 |
| 10 wt% Ru/SnO_2_ | RuO_2_/SnO_2_ | 397 | 10 |
| 20 wt% Ru/SnO_2_ | RuO_2_/SnO_2_ | 384 | 9 |

[a] Temperature at which N_2_O conversion reached 50%.

**Table S2.** Catalytic properties of 5.0 wt% Ru/SnO_2_ as a function of the number of repeated catalytic cycles.

| Catalyst | Repetition number  and/or coexisting gas | *T*_50_ |
| --- | --- | --- |
|  |  | ^[a]^ /°C |
| 5.0 wt% Ru/SnO_2_ | 1 | 395 |
|  | 2 | 402 |
|  | 3 | 412 |
|  | 4 | 417 |
|  | 5 | 427 |
|  | H_2_O | 445 |

[a] Temperature at which N_2_O conversion reached 50%.

**Figure S1.** H_2_-TPR profiles of Ru supported on various metal oxide materials.

**Figure S2.** Ru3d XPS spectra for 5.0 wt% Ru catalysts supported on various oxide materials were obtained.

**Figure S3.** NH_3_-TPD profiles of Ru supported on various metal oxide materials.

**Figure S4.** NO-TPD profiles of Ru supported on various metal oxide materials.

**Figure S5.** PXRD patterns of the 0.5–20 wt% Ru/SnO_2_ catalysts prepared by impregnation, followed by drying and calcination at 600°C for 3 h in air.

**Figure S6.** N_2_O conversion, NO selectivity and mass signal for N_2_O decomposition reaction over 5.0 wt% Ru/SnO_2_. Reaction conditions: 200 ppm of N_2_O, 10% O_2_ and N_2_ balance at 100 cm^3^·min^−1^ (W/F = 5.0 × 10^−4^ g·min·cm^−3^).

**Figure S7.** In situ FTIR spectra of N_2_O adsorbed on 5.0 wt% Ru/SnO_2_. The spectra were measured at 300°C and 400°C in gas feeds of 200 ppm of N_2_O and N_2_ balance.

**Figure S8.** Catalytic activity for the N_2_O decomposition reaction over 5.0 wt% Ru/SnO_2_ as a function of the repetition number, and the effects of water vapour (10% H_2_O). Reaction conditions: 200 ppm of N_2_O, 10% O_2_ and N_2_ balance at 100 m^3^·min^−1^ (W/F = 5.0 × 10^−4^ g·min·cm^−3^).

**Figure S9.** (a) XRD patterns and (b) Ru3d XPS spectra for 5.0 wt% Ru/SnO_2_ before and after N_2_O decomposition reaction.

**Figure S10.** Time-on-stream stability of catalytic activity for the N_2_O decomposition reaction over 5.0 wt% Ru/SnO_2_. Reaction conditions: 200 ppm of N_2_O, 10% O_2_ and N_2_ balance at 100 cm^3^·min^−1^ (W/F = 5.0 × 10^−4^ g·min·cm^−3^), 400°C.
